# Supplementary material for: Unattached kinetochores drive their own capturing by sequestering a CLASP
Source: Nat Commun. 2018 Feb 28;9:886. doi: 10.1038/s41467-018-03108-z (PMC5830412; doi:10.1038/s41467-018-03108-z)
Supplement: Supplementary file 1 — Supplementary Information [file 41467_2018_3108_MOESM1_ESM.pdf]

Supplementary information

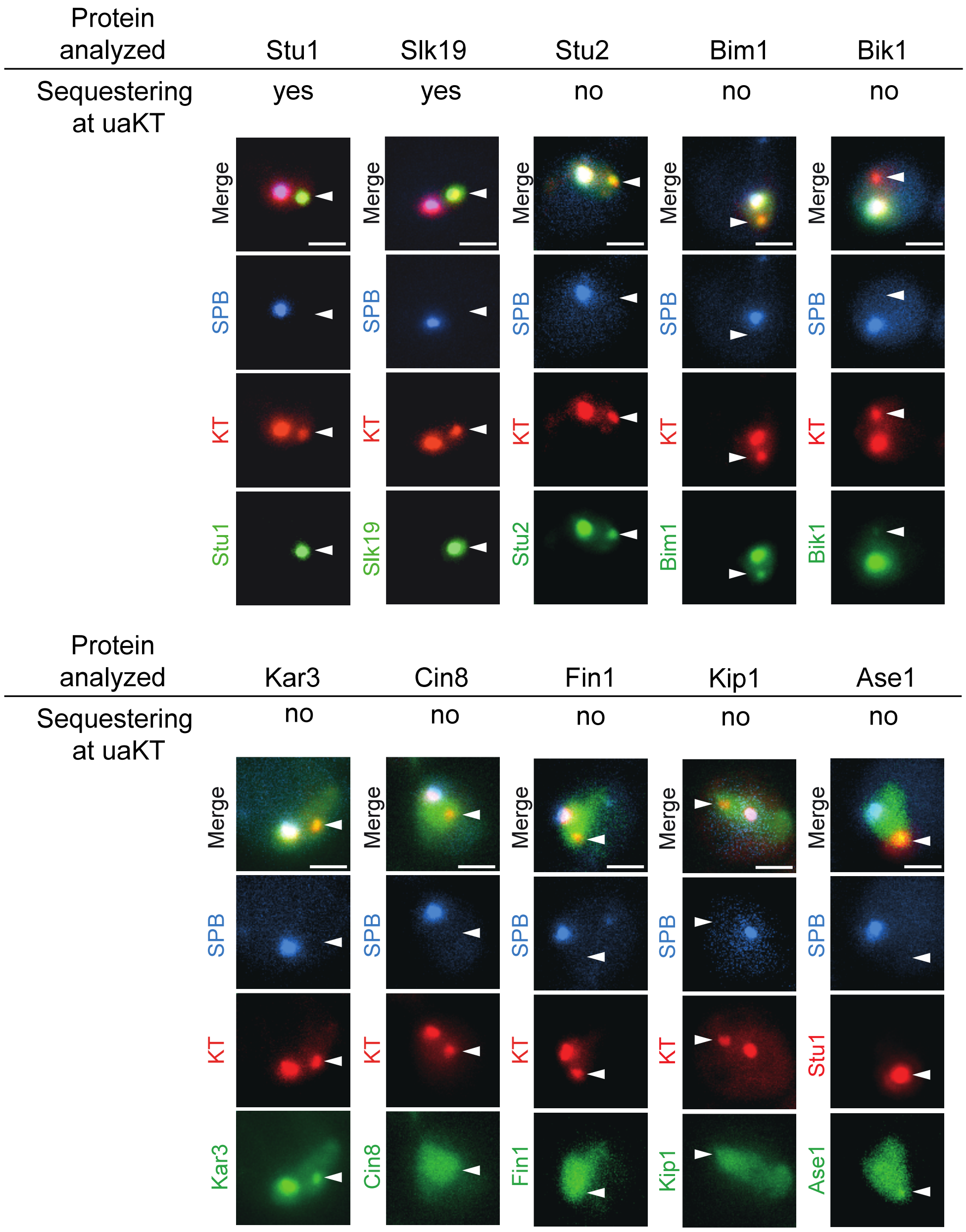

Supplementary Figure 1 In contrast to Stu1 and Slk19, the microtubule-associated proteins Stu2, Bim1, Bik1, Kar3, Cin8, Fin1, Kip1 and Ase1 are not sequestered at uaKTs. Genotypes of the used strains are listed in Supplementary Table 1. Bars, 2μm. White arrowheads mark uaKTs. Cells were analyzed 3-4 h after the release from G1 into nocodazole. The Stu1 and Slk19 phenotypes are as shown in Figure 1 and have been included for comparison. The other phenotypes shown are representative for the indicated proteins. Most notably, no sequestering was observed in n>100 cells in these cases.

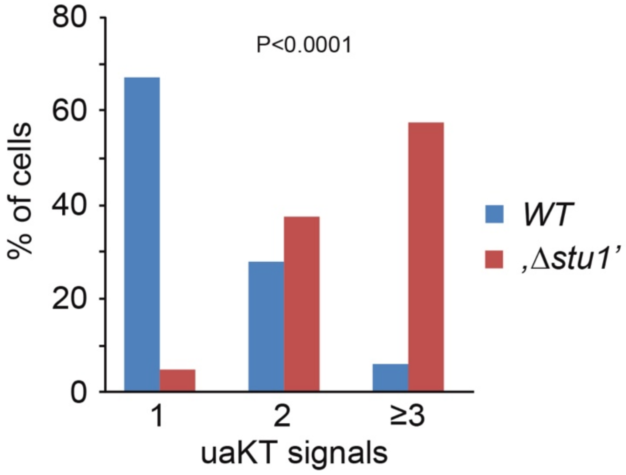

Supplementary Figure 2 Sequestering of Stu1 supports the clustering of uaKTs. Genotypes of the used strains are listed in Supplementary Table 1. Statistics see Supplementary Table 3. Cells with the indicated genetic background were analyzed 3-4 h after the release from G1 into nocodazole (see Fig. 1b and d for phenotypes). Only cells that revealed at least one uaKT cluster were included in the analysis. ‚Δ*stu1*' indicates that Stu1 was depleted.

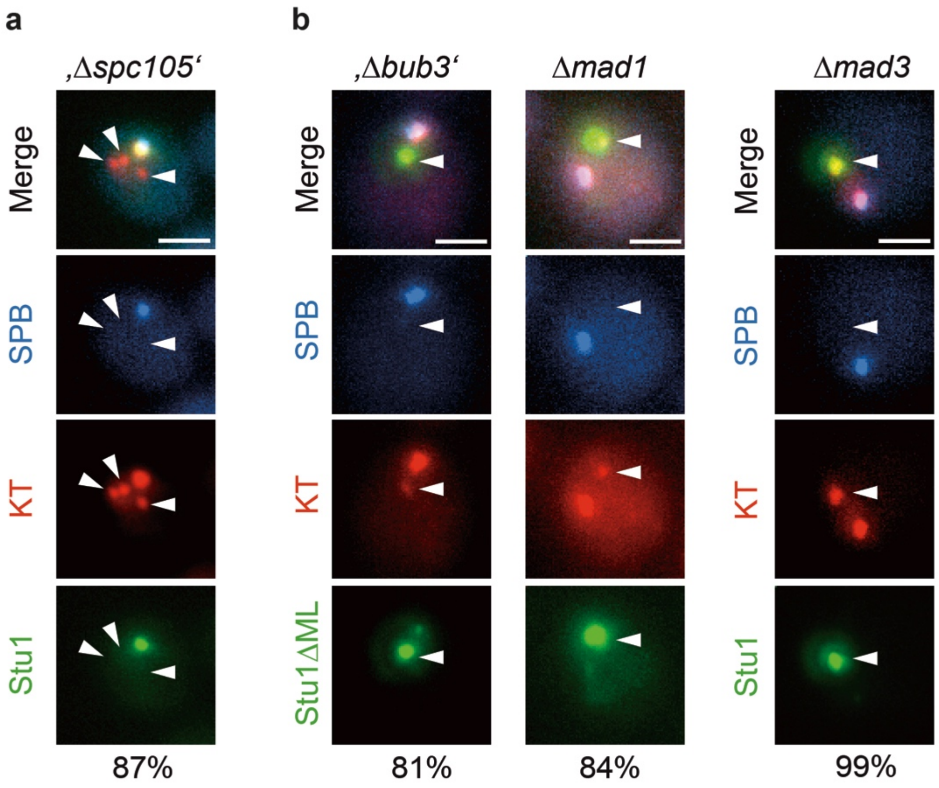

Supplementary Figure 3 Sequestering and localization of Stu1 at uaKTs depends on Spc105 but not on Bub3, Mad1 and Mad3. **(a-b)** Genotypes of the used strains are listed in Supplementary Table 1. SAC-deficient cells can progress into anaphase despite nocodazole treatment. This might affect the results (for instance due to Mps1 inactivation). Therefore, cells with the indicated genetic background were released from G1 into nocodazole while inducing metaphase arrest by Cdc20 depletion. *'Δspc105'* and *'bub3'* indicates that the proteins were depleted after the metaphase arrest. The percentage of cells that revealed the depicted phenotype is indicated. White arrowheads indicate uaKTs. Bars, 2µm. Statistics see Supplementary Table 3. **(a)** Spc105 is essential for Stu1 localization and sequestering at uaKTs. **(b)** Bub3, Mad1 and Mad3 are not essential for Stu1ΔML or Stu1 sequestering. Stu1 in the background was depleted.

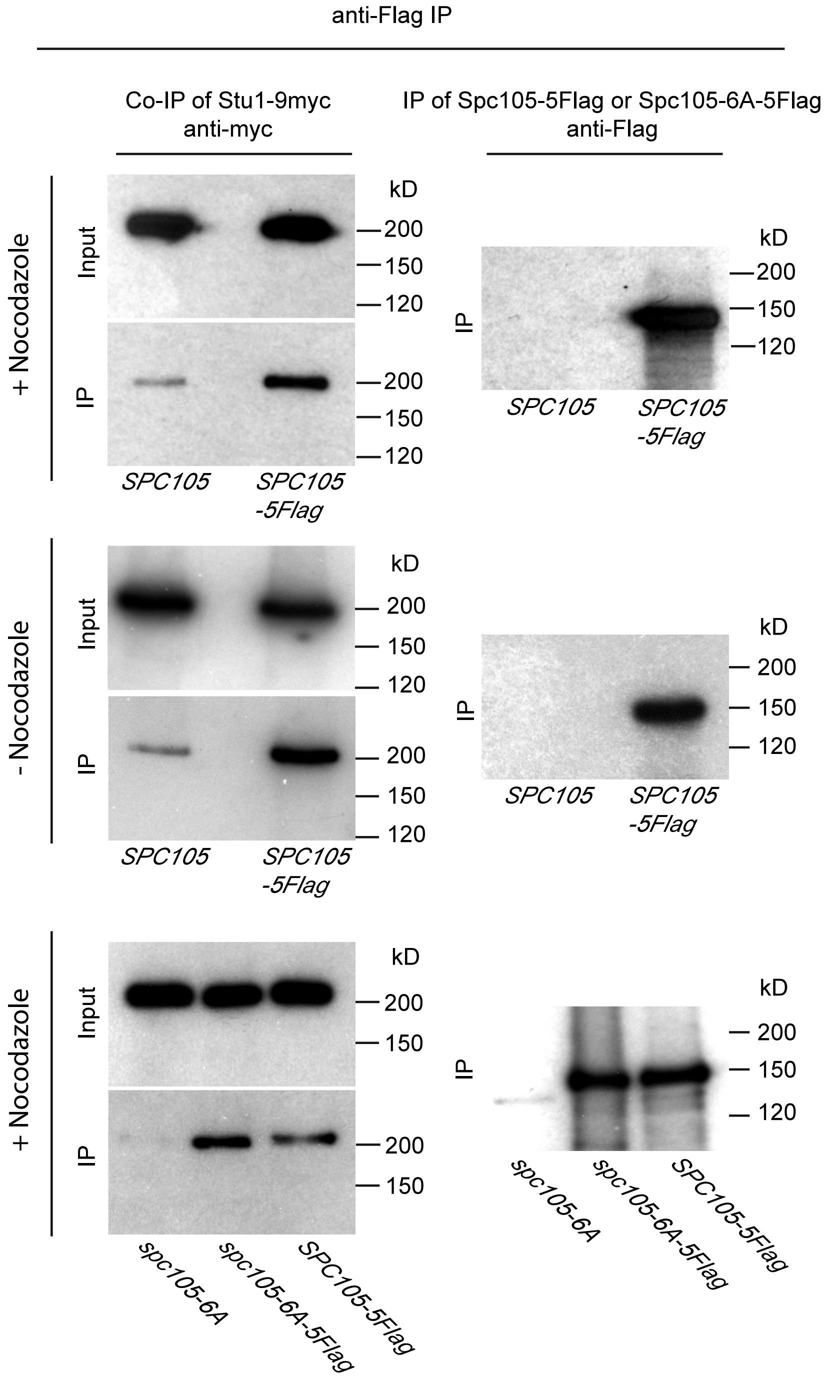

Supplementary Figure 4 Stu1 co-purifies with Spc105 or Spc105-6A in nocodazole-arrested cells and Spc105 in cycling cells. Genotypes of the used strains are listed in Supplementary Table 1. Stu1-9myc and Spc105-5Flag or Spc105-6A-5Flag were coexpressed under endogenous promoters in *S. cerevisiae* and Spc105-5Flag or Spc105-6A-5Flag was affinity purified. Wild type Spc105 in the background of *spc105-6A* cells was depleted. Note that Stu1 resides exclusively at uaKTs in nocodazole-treated *SPC105* WT cells (Fig. 1b) but predominantly at attached KTs in nocodazole-treated *spc105-6A* cells (Fig. 1l) or untreated *SPC105* wildtype cells.

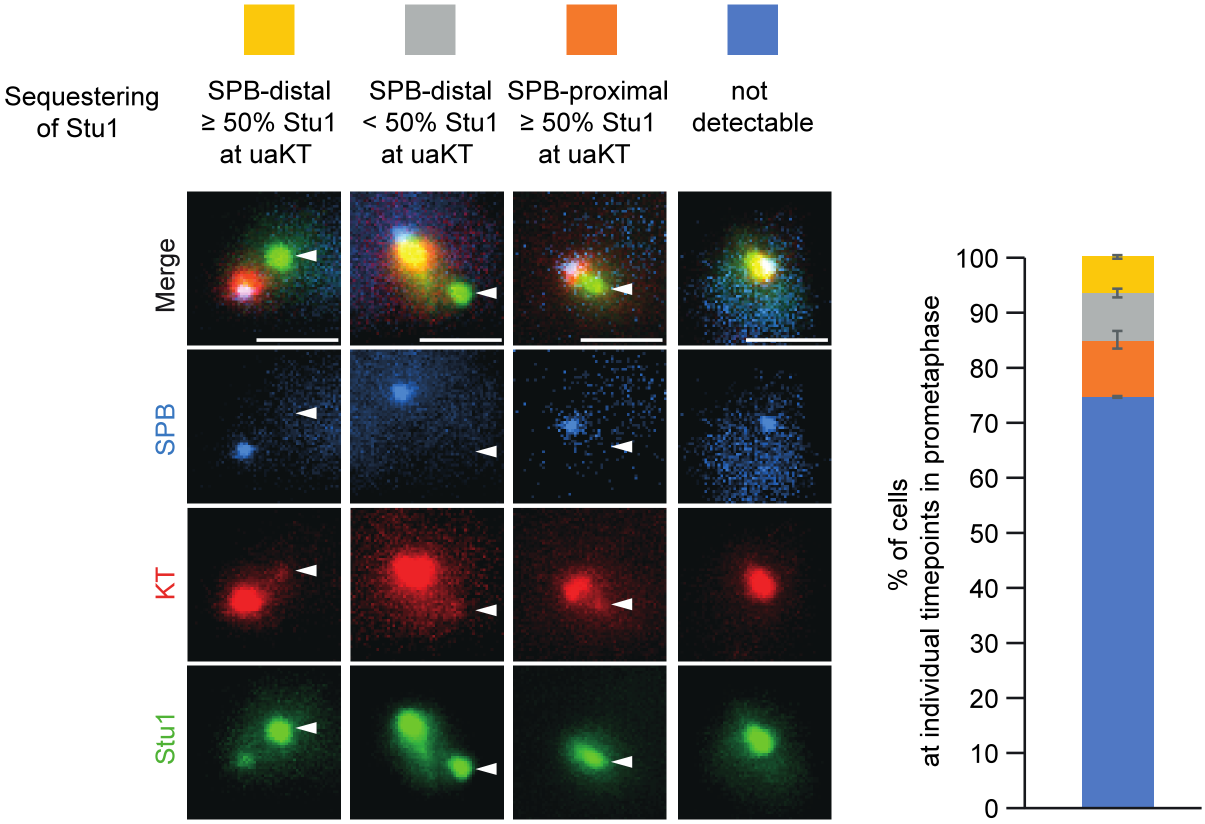

Supplementary Figure 5 Cells in prometaphase exhibit uaKTs that sequester Stu1. The genotype of the used strain is listed in Supplementary Table 1. Bars, 2µm. Cells were analyzed at individual timepoints 45-67min after the release from G1. Only cells with a small bud (diameter ≤1.6µm) were counted. Error bars represent the standard deviation of three independent experiments. White arrowheads mark uaKTs. Statistics see Supplementary Table 3.

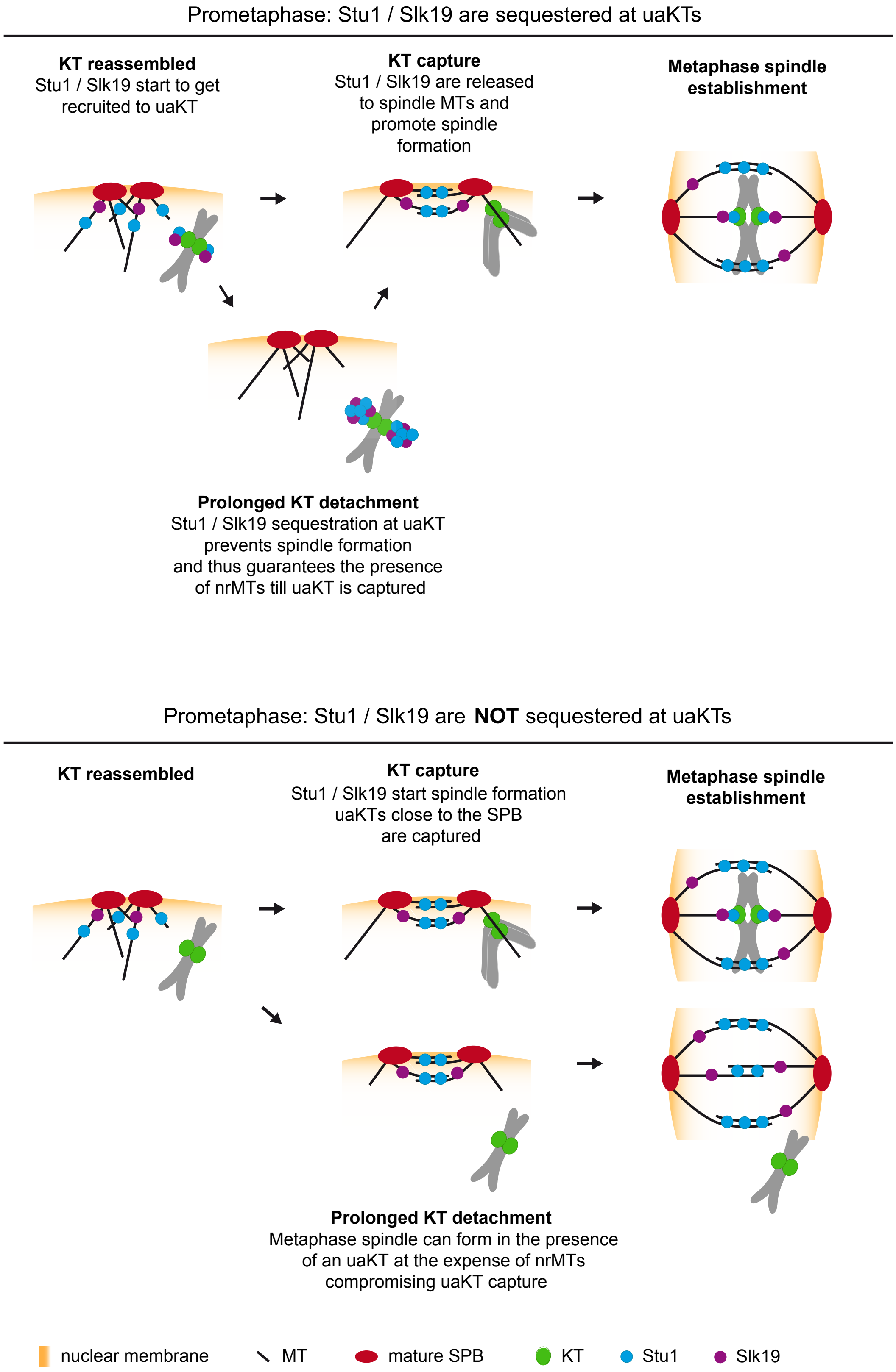

Prometaphase: Stu1 / Slk19 are sequestered at uaKTs
KT reassembled
Stu1 / Slk19 start to get recruited to uaKT
KT capture
Stu1 / Slk19 are released to spindle MTs and promote spindle formation
Metaphase spindle establishment
Prolonged KT detachment
Stu1 / Slk19 sequestration at uaKT prevents spindle formation and thus guarantees the presence of nrMTs till uaKT is captured
Prometaphase: Stu1 / Slk19 are NOT sequestered at uaKTs
KT reassembled
KT capture
Stu1 / Slk19 start spindle formation uaKTs close to the SPB are captured
Metaphase spindle establishment
Prolonged KT detachment
Metaphase spindle can form in the presence of an uaKT at the expense of nrMTs compromising uaKT capture
nuclear membrane
MT
mature SPB
KT
Stu1
Slk19

Supplementary Figure 6 Model depicting the putative role of Stu1 / Slk19 sequestering at uaKTs in prometaphase. Sequestering of Stu1 / Slk19 at prevailing uaKTs prevents spindle formation and thus guarantees the availability of capturing MTs till uaKTs are captured (above). If Stu1 / Slk19 would not be sequestered at prevailing uaKTs, spindle formation would occur in the presence of uaKTs. This would decrease the number and length of capturing MTs and thus compromise the capturing of these uaKTs (below).

Supplementary Table 1 Yeast strains used in this study

Strain name	Relevant Genotype	Figure
YJO1117	*MATa Δsst1 ade2-101ochre trp1-Δ63 leu2-Δ1 ura3-52 his3-Δ200 lys2-801ambre AME1-GFP::kanMX6 STU1-ECFP::HIS3MX6 SPC72-3mCherry::hphNT1*	1b, Supplement 1 and 2
YJO1422	*MATa Δsst1 ade2-101ochre trp1-Δ63 leu2-Δ1 ura3-52 his3-Δ200 lys2-801ambre AME1-GFP::kanMX6 SPC72-3mCherry::hphNT1 SLK19-ECFP::HIS3MX6*	1c, Supplement 1
YJO2718	*MATa Δsst1 ade2-101 ochre::pADH1-OsTIR1-9Myc::ADE2 trp1-Δ63 leu2-Δ1 ura3-52 his3-Δ200 lys2-801ambre NUF2-3mCherry::natNT2 SPC72-ECFP::hphNT1 SLK19-yeGFP::HIS3MX6 STU1-IAA17::kanMX4*	1d Supplement 2
YJO2654	*MATa Δsst1 ade2-101 ochre trp1-Δ63 leu2-Δ1 ura3-52 his3-Δ200 lys2-801 ambre SPC72-ECFP::kanMX4 MTW1-3mCherry::hphNT1 STU1-yeGFP::klTRP1 Δslk19::HIS3MX6*	1e
YVS1651	*MATa Δsst1 ade2-101 ochre trp1-Δ63 leu2-Δ1 ura3-52 his3-Δ200 lys2-801 ambre::CFP-TUB1::LYS2 AME1-1mCherry::hphNT1 stu1Δ(aa995-1180)-EGFP::klTRP1*	1f
YVS2151	*MATa Δsst1 ade2-101 ochre trp1-Δ63 leu2-Δ1 ura3-52 his3-Δ200 lys2-801 ambre SPC72-3mCherry::hphNT1 SLK19-EGFP::klTRP1 stu1Δ(aa995-1180)-CFP::kanMX6*	1g
YJO2717	*MATa Δsst1 ade2-101 ochre::pADH1-OsTIR1-9Myc::ADE2 trp1-Δ63 leu2-Δ1 ura3-52 his3-Δ200 lys2-801 ambre::pSTU1-FLAG-stu1Δ(aa1-260, 1182-1514)-NLS-GFP::LYS2 NUF2-3mCherry::natNT2 SPC72-ECFP::hphNT1 STU1-IAA17::kanMX4*	1h
YJO2716	*MATa Δsst1 ade2-101 ochre::pADH1-OsTIR1-9Myc::ADE2 trp1-Δ63 leu2-Δ1 ura3-52 his3-Δ200 lys2-801 ambre::pSTU1-FLAG-stu1Δ(aa1-260, 997-1514)-NLS-GFP::LYS2 NUF2-3mCherry::natNT2 SPC72-ECFP::hphNT1 STU1-IAA17::kanMX4*	1i
YJO2471	*MATa Δsst1 ade2-101 ochre trp1-Δ63::mps1-as1(M516G)-6HA::klTRP1 leu2-Δ1 ura3-52 his3-Δ200 lys2-801 ambre MTW1-CFP::hphNT1 STU1-3mCherry::natNT2 SPC72-EGFP::kanMX4 Δmps1::HIS3MX6*	1j
YJO2745	*MATa Δsst1 ade2-101 ochre trp1-Δ63::mps1-as1(M516G)-6HA::klTRP1 leu2-Δ1 ura3-52 his3-Δ200 lys2-801 ambre MTW1-CFP::hphNT1 STU1-3mCherry::natNT2 SLK19-yeGFP::kanMX6 Δmps1::HIS3MX6*	1k
YJO2740	*MATa Δsst1 ade2-101 ochre::pADH1-OsTIR1-9Myc::ADE2 trp1-Δ63 leu2-Δ1::spc106-6A (T149A, T172A, T211A, T235A, T284A, T313A)::LEU2 ura3-52 his3-Δ200 lys2-801 ambre NUF2-3mCherry::natNT2 SPC72-yeCFP::hphNT1 STU1-yeGFP::klTRP1 SPC105-IAA17-3HA::HIS3MX6 cdc20::kanMX6-pGAL1-3HA-CDC20*	1l
YJO2818	*Mata Δsst1 ade2-101ochre::P_{ADH1}-OsTIR1-9Myc::ADE2 trp1-Δ63 leu2-Δ1 ura3-52 his3-Δ200 lys2-801ambre::pStu1-FLAG-stu1Δ(aa570-716)-NLS-GFP::LYS2 MTW1-3mcherry::natNT2 SPC72-eCFP::hphNT1 STU1-IAA17::klURA3 SPC105-2xFKBP12 NDC80-3HA-klTRP1 MPS1-FRB::HIS3MX6 Δfpr1::LEU2 tor1-1 cdc20:KANMX6-pGAL1-3HA-CDC2*	1m
YCF2671	*MATa Δsst1 ade2-101 ochre trp1-Δ63 leu2-Δ1 ura3-52::GFP-TUB1::URA3kl his3-Δ200 lys2-801 ambre STU1-3mCherry::natNT2 Δcen5::pGAL1-CEN3-LEU2 ADE1-pURA3-tetR-Cerulean5x::hphNT1 CEN5-tetO2x112-tetOx112::HIS3 cdc20::klTRP1-pMET25-CDC20*	2a, 6a,7c, 8a-j
YME2710	*MATa Δsst1 ade2-101ochre::pADH1-OsTIR1-9Myc::ADE2 trp1-Δ63 leu2-Δ1 ura3-52::GFP-TUB1::URA3kl his3-Δ200 lys2-801 ambre::pSTU1-stu1Δ(aa995-1180)-1mCherry::LYS2 STU1-IAA17::KANMX4 Δcen5::pGAL1-CEN3-LEU2 ADE1-pURA3-tetR-Cerulean5x::hphNT1 CEN5-tetO2x112-tetOx112::HIS3 cdc20::klTRP1-pMET25-CDC20*	2b, 6a
YCF2673	*MATa Δsst1 ade2-101 ochre trp1-Δ63 leu2-Δ1 ura3-52::GFP-TUB1::URA3kl his3-Δ200 lys2-801 ambre STU1-3mCherry::natNT2 Δcen5::pGAL1-CEN3-LEU2 ADE1-pURA3-tetR-Cerulean5x::hphNT1 CEN5-tetO2x112-tetOx112::HIS3 cdc20::klTRP1-pMET25-CDC20 Δslk19::KANMX6*	2c, 6a, 8a-j
YME2902	*MATa Δsst1 ade2-101ochre::pADH1-OsTIR1-9Myc::ADE2 trp1-Δ63 leu2-Δ1 ura3-52 his3-Δ200 lys2-80ambre STU1-3mCherry::natNT2 DAD1-yeGFP::klTRP1 Δcen5::pGAL1-CEN3-LEU2 ADE1-pURA3-tetR-CFP3x::hphNT1 CEN5-tetO2x112::HIS3 cdc20::URA3kl-pMET25-CDC20 MPS1-IAA17-3HA-kanMX4*	2d, 4a-e, 6a
YCF2475	*MATa Δsst1 ade2-101 ochre trp1-Δ63 leu2-Δ1 ura3-52::GFP-TUB1::URA3kl his3-Δ200 lys2-801 ambre STU1-3mCherry::hphNT1 Δcen5::pGAL1-CEN3-LEU2 cdc20::HIS3MX6-pMET25-CDC20*	3a
YCF2601	*MATa Δsst1 ade2-101ochre trp1-Δ63 leu2-Δ1 ura3-52 his3-Δ200 lys2-80ambre STU1-3mCherry::natNT2 DAD1-yeGFP::klTRP1 Δcen5::pGAL1-CEN3-LEU2 ADE1-pURA3-tetR-CFP3x::hphNT1 CEN5-tetO2x112::HIS3 cdc20::URA3kl-pMET25-CDC20*	3b-g, 5a-e
YME2761	*MATa Δsst1 ade2-101ochre::pADH1-OsTIR1-9Myc::ADE2 trp1-Δ63 leu2-Δ1 ura3-52 his3-Δ200 lys2-801 ambre::stu1Δ(aa995-1180)-1mCherry::LYS2 DAD1-yeGFP::klTRP1 STU1-IAA17::KANMX6 Δcen5::pGAL1-CEN3-LEU2 ADE1-pURA3-tetR-Cerulean5x::hphNT1 CEN5-tetO2x112-tetOx112::HIS3 cdc20::natNT2-pMET25-CDC20*	4a-e
YME2719	*MATa Δsst1 ade2-101 ochre trp1-Δ63 leu2-Δ1 ura3-52 his3-Δ200 lys2-801 ambre DAD1-yeGFP::klTRP1 STU1-3mCherry::natNT2 Δcen5::pGAL1-CEN3-LEU2 ADE1-pURA3-tetR-CFP3x::hphNT1 CEN5-tetO2x112::HIS3 cdc20::URA3kl-pMET25-CDC20 Δslk19::KANMX6*	5a-d
YCF2613	*MATa Δsst1 ade2-101ochre::pADH1-OsTIR1-9Myc::ADE2 trp1-Δ63::CFP-TUB1::klTRP1 leu2-Δ1 ura3-52 his3-Δ200 lys2-801 ambre DAD1-yeGFP::hphNT1 STU1-IAA17::KANMX4 cdc20::natNT2-pMET25-CDC20*	5a-f
YME2689	*MATa Δsst1 ade2-101ochre::pADH1-OsTIR1-9Myc::ADE2 trp1-Δ63 leu2-Δ1 ura3-52::GFP-TUB1::URA3-kl his3-Δ200 lys2-801 ambre STU1-IAA17::KANMX4 Δcen5::pGAL1-CEN3-LEU2 ADE1-pURA3-tetR-Cerulean5x::hphNT1 CEN5-tetO2x112-tetOx112::HIS3 cdc20::klTRP1-pMET25-CDC20*	6a, 8a-j
YME2841	*MATa Δsst1 ade2-101ochre trp1-Δ63 leu2-Δ1 ura3-52::GFP-TUB1::URA3kl his3-Δ200 lys2-801ambre STU1-3mCherry::natNT2 SLK19-ECFP-kanMX4 Δcen5::pGAL1-CEN3-LEU2*	7a

	cdc20::HIS3MX6-pMET25-CDC20	
YCF2535	*MATa Δsst1 ade2-101 ochre trp1-Δ63 leu2-Δ1 ura3-52::GFP-TUB1::URA3kl his3-Δ200 lys2-801ambre STU1-3mCherry::natNT2 Δcen5::pGAL1-CEN3-LEU2 ADE1-pURA3-tetR-CFP3x::hphNT1 CEN5-tetO2x112::HIS3 cdc20::klTRP1-pMET25-CDC20*	7b
YME2731	*MATa Δsst1 ade2-101ochre:: pADH1-OsTIR1-9Myc::ADE2 trp1-Δ63 leu2-Δ1 ura3-52::GFP-TUB1::URA3kl his3-Δ200 lys2-801ambre:: pSTU1-STU1-3mCherry-pDAM1-dam1Δ(aa206-344)::LYS2 DAM1-IAA::KANMX4 STU1-IAA17::klTRP1 Δcen5::pGAL1-CEN3-LEU2 ADE1-pURA3-tetR-Cerulean5x::hphNT1 CEN5-tetO2x112-tetOx112::HIS3 cdc20::natNT2-pMET25-CDC20*	8k-l
YME2726	*MATa Δsst1 ade2-101ochre::pADH1-OsTIR1-9Myc::ADE2 trp1-Δ63 leu2-Δ1 ura3-52::GFP-TUB1::URA3kl his3-Δ200 lys2-801ambre::pDAM1-dam1Δ(aa206-344)::LYS2 DAM1-IAA17::KANMX4 STU1-IAA17::klTRP1 Δcen5::pGAL1-CEN3-LEU2 ADE1-pURA3-tetR-Cerulean5x::hphNT1 CEN5-tetO2x112-tetOx112::HIS3 cdc20::natNT2-pMET25-CDC20*	8k-l
YME2782	*MATa Δsst1 ade2-101 ochre::pADH1-OsTIR1-9Myc::ADE2 trp1-Δ63 leu2-Δ1 ura3-52::GFP-TUB1::URA3kl his3-Δ200 lys2-801ambre::pSTU1-STU1-3mCherry-pDAM1-dam1Δ(aa206-344)::LYS2 DAM1-IAA17::kanMX4 STU1-IAA17::klTRP1 Δcen5::pGAL1-CEN3-LEU2 ADE1-pURA3-tetR-Cerulean5x::hphNT1 CEN5-tetO2x112-tetOx112::HIS3 cdc20::natNT2-pMET25-CDC20 Δslk19::ble*	8k-l
YJO1464	*MATa Δsst1 ade2-101ochre trp1-Δ63 leu2-Δ1 ura3-52 his3-Δ200 lys2-801ambre AME1-GFP::kanMX6 SPC72-ECFP::klTRP1 STU2-3mCherry::hphNT1*	Supplement 1
YJO1436	*MATa Δsst1 ade2-101ochre trp1-Δ63 leu2-Δ1 ura3-52 his3-Δ200 lys2-801ambre AME1-GFP::kanMX6 SPC72-ECFP::klTRP1 BIM1-3mcherry::hphNT1*	Supplement 1
YJO1429	*MATa Δsst1 ade2-101ochre trp1-Δ63 leu2-Δ1 ura3-52 his3-Δ200 lys2-801ambre AME1-GFP::kanMX6 SPC72-ECFP::klTRP1 BIK1-3mcherry::hphNT1*	Supplement 1
YJO1417	*MATa Δsst1 ade2-101ochre trp1-Δ63 leu2-Δ1 ura3-52 his3-Δ200 lys2-801ambre AME1-GFP::kanMX6 SPC72-ECFP::klTRP1 KAR3-3mcherrry::hphNT1*	Supplement 1
YJO1479	*MATa Δsst1 ade2-101ochre trp1-Δ63 leu2-Δ1 ura3-52 his3-Δ200 lys2-801ambre AME1-GFP::kanMX6 SPC72-ECFP::klTRP1 CIN8-3mCherry::hphNT1*	Supplement 1
YJO1469	*MATa Δsst1 ade2-101ochre trp1-Δ63 leu2-Δ1 ura3-52 his3-Δ200 lys2-801ambre AME1-GFP::kanMX6 SPC72-ECFP::klTRP1 FIN1-3mCherry::hphNT1*	Supplement 1
YJO1441	*MATa Δsst1 ade2-101ochre trp1-Δ63 leu2-Δ1 ura3-52 his3-Δ200 lys2-801ambre AME1-GFP::kanMX6 SPC72-ECFP::klTRP1 KIP1-3mcherry::hphNT1*	Supplement 1
YJO1399	*MATa Δsst1 ade2-101ochre trp1-Δ63 leu2-Δ1 ura3-52 his3-Δ200 lys2-801ambre ASE1-4GFP::klTRP1 SPC72-eCFP::kanMX4 STU1-3mcherry::hphNT1*	Supplement 1
YJO2708	*MATa Δsst1 ade2-101ochre::pADH1-OsTIR1-9Myc::ADE2 trp1-Δ63 leu2-Δ1 ura3-52 his3-Δ200 lys2-801ambre NUF2-3mCherry::natNT2 SPC72-yeCFP::hphNT1 STU1-yeGFP::klTRP1 SPC105-IAA17-3HA::HIS3 cdc20::kanMX6-PGAL1-3HA-CDC20*	Supplement 3a
YJO2723	*MATa Δsst1 ade2-101ochre::ADH1-OsTIR1-9Myc::ADE2 trp1-Δ63 leu2-Δ1 ura3-52 his3-Δ200 lys2-801ambre::*P_{STU1}*-stu1Δ(aa570-716)-NLS-GFP::LYS2 NUF2-3mCherry::natNT2 SPC72-eCFP::hphNT1 STU1-IAA17::KanMX4 BUB3-IAA17::HIS3 cdc20::TRP1-pGAL1-3HA-CDC20*	Supplement 3b
YJO2727	*MATa Δsst1 ade2-101ochre::*P_{ADH1}*-OsTIR1-9Myc::ADE2 trp1-Δ63 leu2-Δ1 ura3-52 his3-Δ200 lys2-801ambre::*P_{STU1}*-FLAG-stu1Δ(aa570-716)-NLS-GFP::LYS2 NUF2-3mCherry::natNT2 SPC72-eCFP::hphNT1 STU1-IAA17::KanMX4 Δmad1:loxP cdc20::HIS3MX6-pGAL1-3HA-CDC20*	Supplement 3b
YJO2907	*MATa Δsst1 ade2-101ochre trp1-Δ63 leu2-Δ1 ura3-52 his3-Δ200 lys2-801ambre MTW-3mCherry::hphNT1 STU1-yeGFP::klTRP1 SPC72-ECFP::kanMX4 Δmad3::HIS3MX6 cdc20::klURA3-pGAL1-3HA-CDC20*	Supplement 3b
YJO1206	*MATa Δsst1 ade2-101ochre trp1-Δ63 leu2-Δ1 ura3-52 his3-Δ200 lys2-801ambre STU1-9myc::kanMX6*	Supplement 4
YJO2886	*MATa Δsst1 ade2-101ochre trp1-Δ63 leu2-Δ1 ura3-52 his3-Δ200 lys2-801ambre STU1-9myc::kanMX6 SPC105-5FLAG::hphNT1*	Supplement 4
YJO2948	*MATa Δsst1 ade2-101ochre::pADH1-OsTIR1-9Myc::ADE2 trp1-Δ63 leu2-Δ1:pSPC105-spc105-6A(T149A, T172A, T211A, T235A, T284A, T313A)-5FLAG-LEU2 ura3-52 his3-Δ200 lys2-801ambre NUF2-3mcherry::natNT2 SPB72-yeCFP::hphT1 STU1-9myc::klTRP1 SPC105-IAA17-3HA::HIS3*	Supplement 4
YME2441	*MATa Δsst ade2-101ochre trp1-Δ63 leu2-Δ1 ura3-52 his3-Δ200 lys2-801ambre STU1-3mCherry::hphNT1 SPC72-ECFP::kanMX4 NUF2-yeGFP::HIS3MX6*	Supplement 5

Supplementary Table 2 Plasmids used in this study

Plasmid name	Description
pVS1499	*pSTU1-FLAG-stu1Δ(aa570-716)-NLS-GFP in YDpK, ampR*
pJO1598	*pSTU1-FLAG-stu1Δ(aa1-260, 997-1514)-NLS-GFP in YDpK, ampR*
pJO1599	*pSTU1-FLAG-stu1Δ(aa1-260, 1182-1514)-NLS-GFP in YDpK, ampR*
pME1546	*pSPC105-spc105-6A (T149A, T172A, T211A, T235A, T284A, T313A) in pRS305, ampR*
pME1597	*pSTU1-stu1Δ(aa995-1180)-1mCherry in YDp-K, ampR*
pCF1563	*pDAM1-dam1Δ(aa206-344) in YDp-K, ampR*
pME1561	*pSTU1-STU1-3mCherry-pDAM1-dam1Δ(aa206-344) in YDp-K, ampR*
pME1595	*IAA17::klTRP1, ampR*
pVS1453	*pADH1-OsTIR1-9Myc::ADE2, ampR*
pMK1459	*Δcen5::pGAL1-CEN3::LEU2, ampR*
pME1590	*ADE1-pURA-tetR-Cerulean5x::hphNT1, ampR*

Supplementary Table 3 Statistics source data

Fig. 1b	Stu1 sequestered at uaKT	Observed Cells	% of cells	Statistics
WT	99	100	99	

Fig. 1c	Slk19 sequestered at uaKT	Observed Cells	% of cells	Statistics
WT	87	87	100	

Fig. 1d	Slk19 not sequestered but localized at uaKT	Observed Cells	% of cells	Statistics[1] Fisher
‚Δstu1'	91	97	94	$p < 0.0001$

Fig. 1e	Stu1 not sequestered but localized at uaKT	Observed Cells	% of cells	Statistics[1] Fisher
Δslk19	75	85	88	$p < 0.0001$

Fig. 1f	Stu1ΔCL not sequestered but localized at uaKT	Observed Cells	% of cells	Statistics[1] Fisher
stu1ΔCL	73	85	86	$p < 0.0001$

Fig. 1g	Slk19 not sequestered but localized at uaKT	Observed Cells	% of cells	Statistics[1] Fisher
stu1ΔCL	89	89	100	$p < 0.0001$

Fig. 1h	Stu1ΔTOGL1ΔD4 localized at uaKT	Observed Cells	% of cells	Statistics
stu1ΔTOGL1ΔD4	87	100	87	

Fig. 1i	Stu1ΔTOGL1ΔCLΔD4 not localized at uaKT	Observed Cells	% of cells	Statistics[2] Fisher
stu1ΔTOGL1-ΔCLΔD4	103	134	77	$p < 0.0001$

Fig. 1j	Stu1 not localized at uaKT	Observed Cells	% of cells	Statistics[1] Fisher
mps1-as1	97	100	97	$p < 0.0001$

Fig. 1k	Slk19 not sequestered but localized at uaKT	Observed Cells	% of cells	Statistics[1] Fisher
mps1-as1	103	108	95	$p < 0.0001$

Fig. 1l	Stu1 not localized at uaKT	Observed Cells	% of cells	Statistics[1] Fisher
spc105-6A	171	219	78	$p < 0.0001$

Fig. 1m	Stu1 sequestered at attached KT	Observed Cells	% of cells	Statistics[3] Fisher
- Rapamycin	2	95	2	
+ Rapamycin	93	109	85	$p < 0.0001$

Fig. 2a-c. Intensity of Stu1 localization	Observed Cells
WT	33
stu1ΔCL	22
Δslk19	29
‚Δmps1'	20

Fig. 3d,e, MT length (*WT*)	Observed Cells	Observed Timepoints	Statistics[4] Mann-Whitney
No uaKT	87	1275	
uaKT	60	545	p < 0.0001

Fig. 3f. MT number (*WT*)	Observed Cells	Observed Timepoints	Statistics[4] Chi-square
No uaKT	87	1275	
uaKT	60	545	p < 0.0001

Fig. 3g. MT length distribution (*WT*)	Observed Cells	Observed Timepoints	Statistics[4] Chi-square[7]
No uaKT	87	1275	
uaKT	60	545	p < 0.0001

Fig. 4b,c MT length	Observed Cells	Observed Timepoints	Statistics Mann-Whitney
stu1ΔCL with uaKT	58	827	p < 0.0001[5]
‚*Δmps1*‘ with uaKT	87	1174	p < 0.0001[5]
‚*Δmps1*‘ without uaKT	82	1231	p = 0.3789[6]

Fig. 4d. MT number	Observed Cells	Observed Timepoints	Statistics[5] Chi-square
stu1ΔCL with uaKT	58	827	p < 0.0001
‚*Δmps1*‘ with uaKT	87	1174	p < 0.0001

Fig. 4e. MT length distribution	Observed Cells	Observed Timepoints	Statistics[5] Chi-square[7]
stu1ΔCL with uaKT	58	827	p < 0.0001
‚*Δmps1*‘ with uaKT	87	1174	p < 0.0001

Fig. 5b. MT length	Observed Cells	Observed Timepoints	Statistics[1] Mann-Whitney
WT (no uaKT)	202	202	
‚Δstu1’	208	208	p < 0.0001
Δslk19	178	178	p < 0.0001

Fig. 5c. MT number	Observed Cells	Observed Timepoints	Statistics[1] Chi-square
WT (no uaKT)	202	202	
‚Δstu1’	208	208	p < 0.0001
Δslk19	178	178	p < 0.0001

Fig. 5d. MT length distribution	Observed Cells	Observed Timepoints	Statistics[1] Chi-square[7]
WT (no uaKT)	202	202	
‚Δstu1’	208	208	p < 0.0001
Δslk19	178	178	p < 0.0001

Fig. 5e. MT growth rate	Observed Cells	Observed MTs	Statistics[5] Mann-Whitney
WT with uaKT	54	46	
‚Δstu1’	27	41	p = 0.003

Fig. 5e. MT shrinkage rate	Observed Cells	Observed MTs	Statistics[5] Mann-Whitney
WT with uaKT	54	46	
‚Δstu1’	27	41	p = 0.017

Fig. 5e. MT rescue frequency	Observed Cells	Observed MTs	Observed Events
WT with uaKT	54	46	11
‚Δstu1'	27	41	4

Fig. 5e. MT catastrophe frequency	Observed Cells	Observed MTs	Observed Events
WT with uaKT	54	46	53
‚Δstu1'	27	41	44

Fig. 5e. MT without rescue event	Observed Cells	Observed MTs	Statistics[5] Fisher
WT with uaKT	54	46	
‚Δstu1'	27	41	$p = 0.044$

Fig. 6a. Capturing efficiency	Observed Cells
WT	86
Δslk19	66
‚Δstu1'	67
stu1ΔCL	79
stu1ΔCL + STU1	81
‚Δmps1'	104

Fig. 7a Slk19 and Stu1 colocalization after capturing	Observed capturing events	Observed timepoints
	12	23

Fig. 7c Stu1 localization after capturing	Observed capturing events
	86

Fig. 7c Stu1 localization after capturing	S.D. of 2 experiments
Stu1 behind + overtakes KT	1.72
Stu1 behind + does not overtake KT	6.47
Stu1 Co-transport +overtakes KT	4.17
Stu1 Co-transport does not overtake KT	1.72
Stu1 precedes	2.29

Fig. 8a, Outcome of MT interaction	Observed Cells	Observed Capt. Events	Statistics[1] Fisher
WT	127	120	
Δslk19	124	128	$p = 0.0025$
‚Δstu1'	127	147	$p < 0.0001$

Fig. 8d, Rescue after lateral attachment	Observed Cells	Observed Capt. Events	Statistics[1] Fisher
WT	127	138	
Δslk19	124	138	$p = 0.0027$
‚Δstu1'	127	171	$p = 0.0027$

Fig. 8g, Rescue when the MT plus-end reaches the KT	Observed Cells	Observed Capt. Events	Statistics[1] Fisher
WT	127	74	
Δslk19	124	98	$p = 0.15$
‚Δstu1'	127	132	$p = 0.069$

Fig. 8i, MT plus-end reaches KT distant to the SPB	Observed Cells	Observed Capt. Events	Statistics[1] Fisher
WT	127	127	
Δ*slk19*	124	135	p = 0.0068
‚Δ*stu1'*	127	165	p < 0.0001

Fig. 8j, Start of end-on pulling	Observed Cells	Observed Capt. Events	Statistics[1] Fisher
WT	127	28	
Δ*slk19*	124	48	p = 0.0097
‚Δ*stu1'*	127	52	p < 0.0001

Fig. 8k, End-on standstill	Observed Cells	Observed Capt. Events	Statistics[1] Fisher
WT	92	101	
Δ*slk19*	136	153	p < 0.0001
‚Δ*stu1'*	95	102	p < 0.0001

Supp. Fig. 2	Observed Cells	Statistics[1] Chi-square
WT	119	
‚Δ*stu1'*	125	p < 0.0001

Supp. Fig. 3a	Stu1 not sequestered at uaKT	Observed Cells	% of cells	Statistics[1] Fisher
‚Δ*spc105'*	112	129	87	p < 0.0001

Supp. Fig. 3b	Stu1ΔML sequestered at uaKT	Observed Cells	% of cells	Statistics[8] Fisher
‚Δ*bub3'*	113	139	81	p < 0.0001
Δ*mad1*	90	107	84	p < 0.0001

Supp. Fig. 3b	Stu1 sequestered at uaKT	Observed Cells	% of cells	Statistics[8] Fisher
Δ*mad3'*	103	104	99	p < 0.0001

Supp. Fig. 5	Observed cells
Experiment 1	153
Experiment 2	155
Experiment 3	130

Supp. Fig. 5 Stu1 Sequestering	Average % of cells (3 experiments)	S.D. of 3 experiments
SPB-distal, ≥50%	6.12	0.86
SPB-distal, <50%	8.44	1.76
SPB-proximal, ≥50%	11.79	3.33
Not detected	73.65	0.71

[1]Compared to *WT*
[2]Compared to *stu1ΔTOGL1ΔD4*
[3]Compared to '- Rapamycin'
[4]Compared to *WT* (no uaKT)
[5]Compared to *WT* with uaKT
[6]Compared to ‚Δ*mps1'* with uaKT
[7]The MT length groups 0, 0-1, 1-2, 2-3 and >3 μm where used for the test
[8]Compared to ‚Δ*slk19'*
